# Nivolumab Versus Regorafenib in Patients With Hepatocellular Carcinoma After Sorafenib Failure

**DOI:** 10.3389/fonc.2021.683341

**Published:** 2021-05-31

**Authors:** Yuan-Hung Kuo, Yi-Hao Yen, Yen-Yang Chen, Kwong-Ming Kee, Chao-Hung Hung, Sheng-Nan Lu, Tsung-Hui Hu, Chien-Hung Chen, Jing-Houng Wang

**Affiliations:** ^1^ Division of Hepatogastroenterology, Department of Internal Medicine, Kaohsiung Chang Gung Memorial Hospital and Chang Gung University College of Medicine, Kaohsiung, Taiwan; ^2^ Division of Hematology-Oncology, Department of Internal Medicine, Kaohsiung Chang Gung Memorial Hospital and Chang Gung University College of Medicine, Kaohsiung, Taiwan

**Keywords:** hepatocellular carcinoma, nivolumab, regorafenib, sorafenib, systemic therapy

## Abstract

**Background:**

Nivolumab and regorafenib are approved second-line therapies for patients with hepatocellular carcinoma (HCC) after sorafenib failure. This study compared the effectiveness of nivolumab and regorafenib following sorafenib.

**Methods:**

We retrospectively enrolled HCC patients who had undergone nivolumab or regorafenib after sorafenib failure. Treatment response, treatment-related adverse events (TRAE) and clinical outcomes of study patients were recorded and analyzed.

**Results:**

A total of 90 patients (male/female: 67/23, mean age: 63 years) were enrolled, including 32 patients in the Nivolumab group and 58 patients in the Regorafenib group. The Nivolumab group had better objective response rates (16% *vs* 6.4%) and disease control rates (44% *vs* 31.9%) than the Regorafenib group, but there was no statistical difference. The comparison of time to progression (3.0 months *vs* 2.6 months, p=0.786) and overall survival (OS) (14 months *vs* 11 months, p = 0.763) between Nivolumab and Regorafenib groups were also insignificant. Regarding number of TRAE incidences, the Nivolumab group was significantly lower than the Regorafenib group (37.5% *vs* 68%). After cession of nivolumab/regorafenib, 34 patients (37.8%) (Nivolumab group/Regorafenib group: 11/23) could afford the following therapies. Concerning sequential systemic therapies, 17 patients (18.9%) received third-line therapy, whereas six patients (6.7%) could move to fourth-line therapy. In multivariable analysis, patients who achieved disease control were associated with improved OS (hazard ratio, 0.18; 95% confidence interval, 0.07–0.46; p<0.001) after adjusting Child-Pugh class and post-treatment.

**Conclusions:**

After sorafenib failure, using nivolumab or regorafenib both illustrated promising treatment outcomes.

## Introduction

Hepatocellular carcinoma (HCC) is the fifth most common cancer and the third leading cause of cancer-related mortality worldwide, accounting for approximately 700,000 deaths annually (1). It is a troublesome tumor with poor prognosis because of frequent late diagnoses with advanced stage, limiting the potential for effective locoregional therapies such as hepatic resection, radiofrequency ablation (RFA) or transarterial chemoembolization (TACE), etc. Hence, systemic therapy is the main therapeutic modality for advanced HCC (2). Sorafenib, the first approved agent for systemic therapy of advanced HCC, is a multi-targeted tyrosine kinase inhibitor (mTKI) that can target several protein receptors such as vascular endothelial growth factor receptor (VEGFR), or platelet-derived growth factor receptor (PDGFR) to impair vascular angiogenesis as well as block several cell signaling pathways such as Raf-1, B-Raf, and kinase activity in the Ras/Raf/MEK/ERK signaling pathways to inhibit tumor proliferation (3). Approval of sorafenib is according to two randomized, double-blind, phase III clinical trials, where sorafenib significantly improved overall survival (OS) in patients with advanced HCC compared with placebo (4, 5); however, the progression of other first-line or following second-line systemic therapies for advanced HCC was disappointing, until 2017, when two second-line agents, regorafenib and nivolumab, have since demonstrated their therapeutic effectiveness for the treatment of HCC (6, 7). The phase III RESORCE study demonstrated that sequential administration of sorafenib followed by that of regorafenib extended patient survival (median survival time: 26.0 months for sorafenib–regorafenib *vs*. 19.6 months for sorafenib–placebo) (6). In addition, regorafenib also prolonged progression-free survival (PFS) compared with placebo (3.1 months *vs* 1.5 months, p<0.001). The recent development of cancer immunotherapies using immune checkpoint inhibitors (ICIs) targeting cytotoxic T-lymphocyte-associated protein-4 (CTLA-4) and anti-programmed cell death protein-1 (PD-1) has dramatically changed the landscape of cancer therapy and prolonged the survival of patients with different malignancies (8). Studies evaluating anti-PD-1/PD-L1 monoclonal antibodies as single agents in pre-treated patients with advanced HCC showed encouraging results (9, 10). Indeed, the blockage of PD-1/PD-L1 expression and tumor-infiltrating lymphocytes (TIL) in patients with HCC leads to the discontinuation of immunosuppressive effect by the cancer cells and therefore reactivates cytotoxic T-cells to identify and eradicate the cancer cells (11). Nivolumab was the first second-line treatment for patients with advanced HCC to be approved, and based on the phase I/II Checkmate 040 study (7), the objective response rate (ORR) was 20%, the disease control rate (DCR) was 64%, and PFS was 4.1 months for patients after sorafenib failure. Although regorafenib and nivolumab both showed significant therapeutic efficacy compared with placebo, which systemic therapy should be applied following sorafenib for patients with advanced HCC was still a critical issue in real clinical practice. Consequently, this study aimed to appraise therapeutic efficacy and safety of two second-line therapies, regorafenib and nivolumab, for patients with advanced HCC after sorafenib failure.

## Patients and Methods

### Patients

This retrospective study included patients with unresectable HCC in intermediate or advanced stages receiving regorafenib or nivolumab in our institute, Kaohsiung Chang Gung Memorial Hospital, from July 2016 until December 2019. HCC diagnosis was confirmed by pathologic identification or dynamic imaging of abdominal computed tomography (CT) or magnetic resonance imaging (MRI) based on international guidelines. The inclusion criteria were 1) unresectable HCC in intermediate or advanced stage; 2) receiving regorafenib or nivolumab after sorafenib failure; and 3) Child-Pugh class A or B. Patients were excluded if they had received prior systemic therapy other than sorafenib, had unclear history of sorafenib treatment, were concurrent with other malignancies, were Child-Pugh class C, or had become lost to follow-up after treatment. Those patients with treatment duration longer than 6 months between sorafenib cessation and regorafenib or nivolumab initiation were also excluded. This study protocol was approved by the Research Ethics Committee of Chang Gung Memorial Hospital (IRB No: 202100227B0).

### Treatment Option

After sorafenib failure, using regorafenb or nivolumab was based on the decision of clinicians and the wishes of patients. Regorafenib was administered orally 160 mg once daily for the first 3 weeks of each 4-week cycle, whereas nivolumab was prescribed intravenously at a dose of 3 mg/kg every 2 weeks. The dosage of regorafenib or nivolumab was adjusted clinically according to the severity of treatment related adverse events (TRAE). The patients in both groups received radiologic assessment by CT or MRI every 2 to 3 months. Treatment with regorafenib or nivolumab was terminated with the occurrence of tumor progression, liver function deterioration, intolerable adverse events or death.

### Treatment Outcome

Treatment outcomes were recorded and analyzed, which included OS, meaning the time from treatment initiation to death; PFS, meaning the time from treatment initiation to disease progression or death; time to progression (TTP), meaning the time from treatment initiation to disease progression; ORR, meaning patients achieved complete response (CR) or partial response (PR); and DCR, meaning patients achieved CR, PR or stable disease status (SD). Radiologic response was assessed based on the modified Response Evaluation Criteria in Solid Tumors (mRECIST) (12). TRAE and disease progression were identified from the review of medical records.

### Statistical Analysis

All patients were followed up till the last date of visit, death, or the end of December 2020. To compare values between the two groups, chi-squared tests were applied to analyze categorical variables, while Student’s *t*-test was used for continuous variables. Quantitative variables were expressed with mean ± SD or median with a range. The objective response and disease control rates in both groups were compared using the Cochran-Mantel-Haenszel test. OS and TTP were analyzed using the Kaplan-Meier method with a log-rank test, while univariate and multivariate analyses were performed using Cox proportional hazards regression models. All *P*-values of *<* 0.05 by two-tailed test were considered significant, with statistical analysis carried out using SPSS 22 software (SPSS Inc., Chicago, IL).

## Results

### Clinical Characteristics

**Figure 1:**
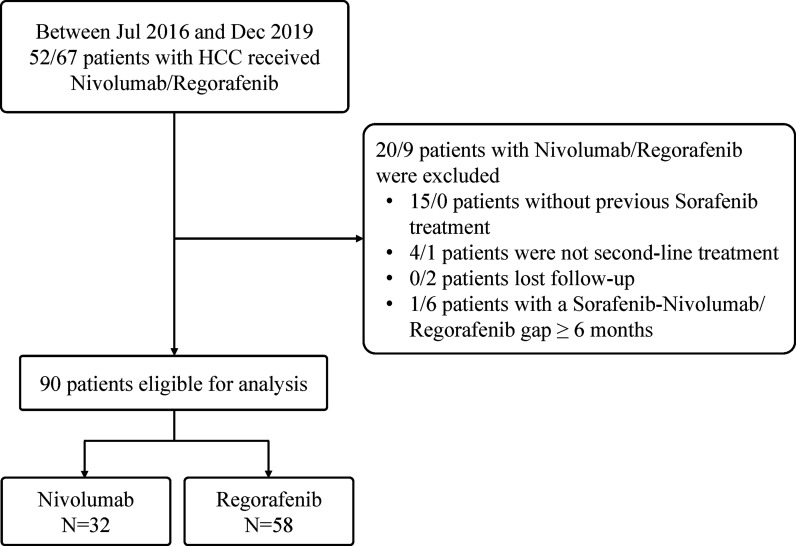
Flow chart of study population.

A total of 119 patients with unresectable HCC in intermediate or advanced stages who received regorafenib (n = 67) or nivolumab (n = 52) therapy between July 2016 and December 2019 in our institute were initially evaluated. Among them, 29 patients were excluded, including 15 patients with nivolumab not receiving previous sorafenib treatment, two patients with regorafenib lost to follow-up, four patients with nivolumab as well as one patient with regorafenib having received more than one systemic therapy before, and one patient with nivolumab as well as 6 patients with regorafenib having a treatment gap longer than 6 months between sorafenib cessation and nivolumab or regorafenib initiation. Finally, 90 patients (75.6%) after sorafenib failure were finally recruited in the study (**Figure 1**), with **Table 1** showing the characteristics of 58 patients included in the Regorafenib group and 32 patients in the Nivolumab group. The mean age of these patients was 63 years and 74.4% were male. Among them, 51% patients had hepatitis B virus (HBV) infection and 37.8% had hepatitis C virus (HCV) infection. Additionally, most patients were Child-Pugh class A. Ten patients (11.1%) received combination therapy with regorafenib or nivolumab including two resections of extra-hepatic tumor, two RFA, two TACE and four RTO, respectively. After cessation of regorafenib or nivolumab, 34 patients (37.8%) still afforded following therapies. Concerning sequential systemic therapies, 17 patients (18.9%) received third-line therapy including one atezolizumab plus bevacizumab, two sorafenib, three pembrolizumab, five nivolumab, and six lenvatinib, respectively. Six patients (6.7%) could move to fourth-line therapy including one atezolizumab plus bevacizumab, two nivolumab, and three lenvatinib, respectively.

**Table 1:** Baseline characteristics of the study population by treatment.

Variables, n (%) or mean ± S.D	Total, N=90	Nivolumab group, N=32	Regorafenib group, N = 58	*P*-value
Age (years)	63 ± 10.4	62.2 ± 10.1	63.4 ± 10.7	0.601
Male sex (%)	67 (74.4)	23 (71.9)	44 (75.9)	0.678
HCC etiology				0.226
HBV	46 (51)	18 (56.3)	28 (48.3)	
HCV	34 (37.8)	11 (34.4)	23 (39.7)	
HBV+HCV	3 (3.2)	2 (6.2)	1 (1.7)	
Non-HBV, non-HCV	7 (7.8)	1 (3.1)	6 (10.3)	
Child-Pugh class				0.01
A	80 (88.9)	25 (78.1)	56 (96.6)	
B	10 (11.1)	7 (21.9)	2 (3.4)	
BCLC stage				0.989
B	14 (15.6)	5 (15.6)	9 (15.5)	
C	76 (84.4)	27 (84.4)	49 (84.5)	
EHM	50 (55.5)	18 (56.3)	32 (55.2)	0.922
Lung	22 (24.4)	9 (28.1)	13 (22.4)	
Lymph node	12 (13.3)	4 (12.5)	8 (13.8)	
Bone	10 (11.1)	4 (12.5)	6 (10.3)	
Others	10 (11.1)	1 (3.1)	9 (15.5)	
MVI*	37 (41.1)	14 (43.8)	23 (39.7)	0.705
VP3	20 (22.2)	6 (18.8)	14 (24.1)	
VP4	17 (18.9)	8 (25)	9 (15.5)	
Tumor size ≥ 6 cm,	18 (20)	10 (33.3)	8 (13.8)	0.031
AST, U/L	82.2 ± 75.4	104.6 ± 89.5	69.9 ± 63.9	0.058
ALT, U/L	59.3 ± 45.3	67.9 ± 45.8	54.5 ± 44.7	0.184
Albumin, g/dL	3.8 ± 0.5	3.6 ± 0.6	3.9 ± 0.4	0.025
Total bilirubin, mg/dL	1.2 ± 0.8	1.5 ± 1.1	1.1 ± 0.4	0.043
Platelet count, ×10^9^/L	142.8 ± 82.8	156.1 ± 99.8	135.7 ± 71.9	0.32
INR	1.06 ± 0.19	1.03 ± 0.3	1.07 ± 0.9	0.403
AFP, ng/mL	7177.3 ± 18321	8348.4 ± 21446	6531.2 ± 16725	0.677
Duration of Sorafenib	2.87 ± 1.99	4.90 ± 2.89	2.75 ± 1.86	<0.001

AFP, alpha fetoprotein; ALBI grade, albumin-biliribin grade; ALT, alanine transaminase; AST, aspartate aminotransferase; BCLC, Barcellola Clinic Liver Cancer; CI, confidence interval; EHM, extra-hepatic metastasis; HBV, hepatitis B virus; HCC, hepatocellular carcinoma; HCV, hepatitis C virus; INR, international ratio; MVI, macro-vascular invasion.

*VP3: Tumor invasion into left portal vein or right portal vein; VP4: Tumor invasion into bilateral portal vein and/or main portal vein.

### Tumor Characteristics

Totally, 84.4% of patients had HCC in BCLC stage C, 41.1% of patients had tumors with macrovascular invasion (MVI), and 55.5% of patients had tumor spread outside the liver (**Table 1**). In HCC patients with MVI, 45.9% were VP4 (tumor invasion into bilateral portal vein and/or main portal vein) whereas 54.1% were VP3 (tumor invasion into left or right portal vein). Regarding HCC patients with extrahepatic metastasis, the top three spreading sites were lung (24.4%), lymph node (13.3%) and bone (11.1%). In addition, 20% of patients had tumor burden larger than 6 cm in diameter.

### Treatment Response

In the Regorafenib group, 47 (81.1%) patients received follow-up dynamic images for the evaluation of treatment response (**Table 2**). Among them, 4.3% of patients achieved CR, 2.1% had PR, 25.5% maintained SD, and 68.1% had progressive disease (PD). ORR was 6.4%, whereas DCR was 31.9%. The duration of regorafenib durability was 5.9 months (range, 1.6–27.33 months). With regar to the Nivolumab group, among 25 patients (78.1%) with following dynamic images, 16% obtained PR, 28% kept SD, and 56% had PD. ORR was 16% and DCR was 44%. The duration of nivolumab durability was 5.8 months (1.8–12.22 months).

**Table 2:** Tumor response in the study population by treatment*.

Variables, n (%) or median (range)	Nivolumab group, N=32	Regorafenib group, N=58
Treatment response evaluation, n(%)	25 (78.1)	47 (81.1)
Complete response	0	2 (4.3)
Partial response	4 (16)	1 (2.1)
Stable disease	7 (28)	12 (25.5)
Progression disease	14 (56)	32 (68.1)
Objective response rate#	16%	6.4%
Disease control rate#	44%	31.9%
Durability, month	5.8 (1.8–12.2)	5.9 (1.6–27.33)
Death	17 (53.1)	28 (48.3)

*Treatment response based on those who received image evaluation including Computer tomography or Magnetic resonance image.

#The comparison of objective response rate (p=0.190) and disease control rate (p=0.309) between two groups was not statistically different.

Two patients in the Regorafenib group achieved CR, both were male, had HBV-related HCC, durable and continued regorafenib use till the end of study observation. One patient experienced sorafenib for 3.6 months and then used regorafenib under the criteria of lung metastasis. Another patient experienced sorafenib for 9.3 months and then received regorafenib due to VP3 invasion. OS of sorafenib-regorafenib sequential therapy in the two patients was 16.4 months and 28.7 months respectively.

### Overall Survival

**Figure 2:**
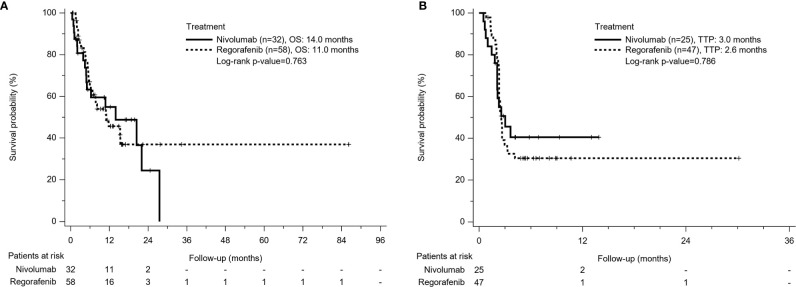
Kaplan-Meier survival curves of treatment outcome including **(A)** overall survival (OS) and **(B)** time to progression (TTP) between Nivolumab group and Regorafenib group. The comparison of OS and TTP in both groups was not different.

A total of 45 patients (50%) died during the follow-up period, including 28 deaths (48.3%) in the Regorafenib group and 17 deaths (53.1%) in the Nivolumab group. From the beginning of sorafenib use, OS was 17.3 months in the Regorafenib group and 21.9 months in the Nivolumab group respectively (p = 0.966). The mean duration of sorafenib use was 2.87 months, which was longer in the Nivolumab group than in the Regorafenib group (4.9 months *vs* 2.75 months, p<0.001). From the time of regorafenib or nivolumab commencement, OS seemed to be longer in the Nivolumab group than in the Regorafeib group, but the comparison was insignificant (14 months *vs* 11 months, p=0.763) (**Figure 2A**).

### Time to Progression

Among patients with radiologic assessment, tumor progression was observed in 68.1% of the Regorafenib group and 56% of the Nivolumab group. The TTP between both groups was not significantly different (2.6 months in the Regorafenib group *vs* 3.0 months in the Nivolumab group, p = 0.786) (**Figure 2B**).

### Factors Associated With Overall Survival

**Figure 3:**
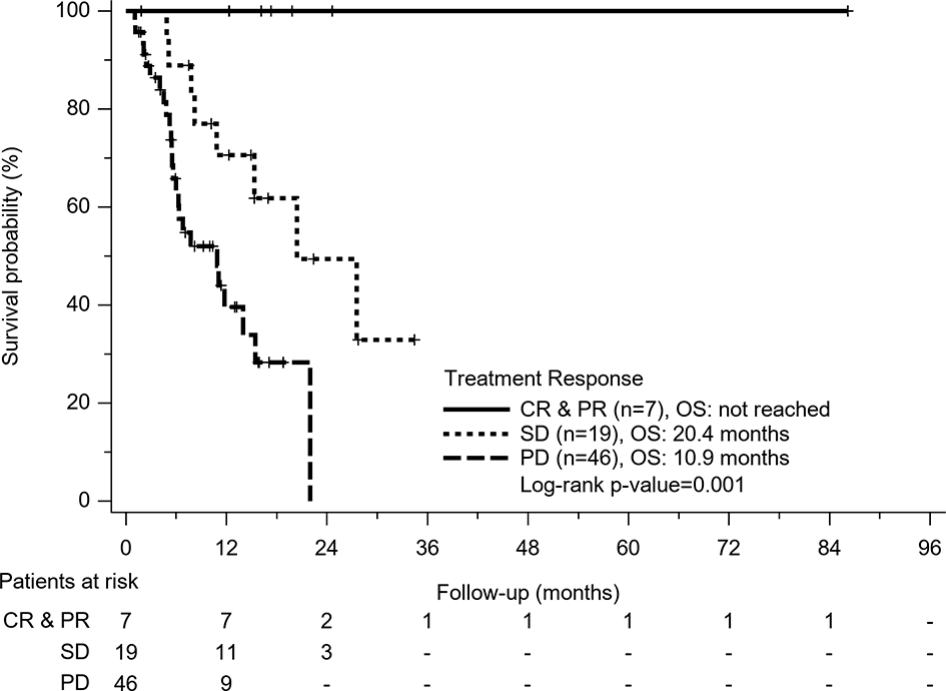
Kaplan-Meier survival curves of treatment response in all patients. Patients who obtained complete response (CR) or partial response (PR) had obvious survival benefits than patients with stable disease (SD) and patients with progression disease (PD) (p=0.001).

In Cox regression model of univariate analysis, older age, poorer liver function reserve, higher AFP level, tumor with macrovascular invasion (MVI), having no disease control, no combination therapy with regorafenib or nivolumab, and no post-regorafenib or nivolumab therapy were independent risk factors associated with mortality (**Table 3**). In multivariate analysis, disease control was a significant predictor of overall survival (hazard ratio: 0.18, 95% confidence interval: 0.07–0.46, p<0.001) after adjustment of Child-Pugh class and post-treatment after regorafenib or nivolumab failure. Different treatment agents using regorafenib or nivolumab did not contribute to overall survival, whether for univariate or multivariate analysis. According to different treatment response, patients obtaining CR or PR had obvious survival benefits (median OS: not reached) than patients with SD (OS: 20.4 months) and patients with PD (OS: 10.9 months) (p=0.001) (**Figure 3**).

**Table 3:** Univariate and multivariate Cox regression analyses for overall survival.

Variables	Comparison	Univariate analysis			Multivariate analysis		
		H.R	95% CI	*P*-value	H.R	95% CI	*P*-value
Age, years	Increase per year	0.96	0.93–0.99	0.009			
Sex	Male *vs*. Female	1.064	0.51–2.11	0.915			
HBV	Yes *vs*. No	0.78	0.42–0.14	0.427			
HCV	Yes *vs* No	1.37	0.73–2.56	0.325			
Child-Pugh class	B *vs* A	4.08	1.69–9.84	0.002	3.4	1.11–10.66	0.033
BCLC stage	C *vs* B	0.92	0.41–2.06	0.829			
EHM	Yes *vs* No	0.75	0.41–1.37	0.349			
MVI	Yes *vs* No	2.24	1.23–4.07	0.009			
AFP ≥ 200 ng/ml	Yes *vs* No	2.03	1.13–3.68	0.019			
Disease control	Yes *vs* No	0.24	0.1–0.57	0.001	0.18	0.07–0.46	<0.001
Combine treatment	Yes *vs* No	0.16	0.04–0.68	0.013			
Post treatment	Yes *vs* No	0.39	0.2–0.75	0.005	0.27	0.12–0.61	0.001
Treatment option	Nivo *vs*. Rego	1.1	0.6–2.01	0.763			

AFP, alpha fetoprotein; BCLC, Barcellola Clinic Liver Cancer; CI, confidence interval; EHM; extra-hepatic metastasis; HBV, hepatitis B virus; HCV, hepatitis C virus; HR, hazard ratio; MVI, macro-vascular invasion.

### Treatment Safety

**Table 4** indicates that sixty-eight percent of patients in the Regorafenib group had treatment-related adverse events, and like sorafenib, the most frequent related adverse event was hand-to-food skin reaction (HFSR), which occurred in 23.8% of patients. Other TRAE with incidence over 10% were diarrhea, fatigue and elevated ALT in descending order of frequency. In addition, six patients (10.2%) had TRAE with severity more than grade 3 requiring permanent cessation of regorafenib, including one HFSR, two with fatigue, and three with hyperbilirubinemia. In the Nivolumab group, 37.5% of patients had any TRAE, including 12.1% with fatigue, 9.3% with dermatitis, and 6.2% with hyperbilirubinemia. Only two patients (6.2%) had severe TRAE over grade 3, and both presented with hyperbilirubinemia. The Nivolumab group was significantly lower than the Regorafenib group (37.5% *vs* 68%, p=0.006).

**Table 4:** Treatment related adverse events (TRAE) in the study population by treatment.

Variables	Nivolumab group (n = 32)		Regorafenib group (n = 58)	
	Any, n (%)	Grade ≥ 3, n (%)	Any, n (%)	Grade ≥ 3, n (%)
Total patients with TRAE*	12 (37.5)	2 (6.2)	40 (68)	6 (10.2)
Hand foot skin reaction, n (%)	0	0	14 (23.8)	1 (1.7)
Diarrhea, n (%)	1 (3.1)	0	7 (11.9)	0
Fatigue, n (%)	4 (12.4)	0	6 (10.2)	2 (3.4)
Elevated AST, n (%)	0	0	6 (10.2)	0
Decreased appetite, n(%)	1(3.1)	0	4 (6.8)	0
Dermatitis, n (%)	3 (9.3)	0	4 (6.8)	0
Elevated T-bil, n (%)	2 (6.2)	2 (6.2)	4 (6.8)	3 (5.1)
Paresthesia, n (%)	1 (3.1)	0	0	0
Hypertension, n (%)	0	0	1 (1.7)	0
Hoarseness, n (%)	0	0	1 (1.7)	0
Pruritus, n (%)	0	0	1 (1.7)	0

AST, aspartate aminotransferase; T-bil, total bilirubin; TRAE, treatment related adverse event.

*The comparison of total patients with TRAE between two groups was significant different (p=0.006).

## Discussion

Nivolumab, an immune checkpoint inhibitor that blocks PD-1, has recently become one of the effective treatment options for many malignancies, including non-small-cell lung cancer, melanoma, renal cell carcinoma, HCC, and the like (7, 13–15). Although there was no significant difference of OS in the CheckMate-459 trial, being the phase III study of nivolumab versus sorafenib as first-line systemic therapy in advanced HCC, nivolumab still demonstrated survival benefit for patients with radiologic response (16). In addition, in the previous CheckMate-040 trial, a phase I/II uncontrolled trial, nivolumab showed durable treatment response and prolonged long-term survival for patients in advanced stage after sorafenib failure (7). Among the total population of 182 patients, there was a promising ORR of 14%, a CR of 3%, and a DCR of 55%, with a 12-month survival rate of 55%. The current study also had a treatment response of nivolumab with an ORR of 16% and a DCR of 44%. In addition, median OS of Nivolumab in the current study was 14 months, equivalent to that of the CheckMate-040 trial (7). It seems that nivolumab monotherapy shows a promising treatment response and an improved survival outcome as second-line therapy for patients with advanced HCC, regardless of being in a clinical trial or in clinical real-world settings.

Regorafenib is an orally administered TKI that is structurally similar to sorafenib but with additional blockage of fibroblast growth factor receptor pathway (17, 18). Therapeutic efficacy of regorafenib has been approved by the phase III RESCORCE trial (6) that included 573 patients with advanced HCC and Child-Pugh class A who tolerated sorafenib but with tumor progression. Under the 2:1 randomized assignment to regroafenib or placebo, regorafenib significantly improved median OS compared with placebo (10.6 months *vs* 7.8 months; HR: 0.62, P<0.0001). Moreover, regorafenib also had prolonged. TTP (3.1 months *vs* 1.5 months, p < 0.0001), improved ORR (10.6% *vs* 4.1%, p=0.0047) and DCR (65.2% *vs* 36.1%, p<0.0001) in comparison with placebo. Therefore, current international HCC treatment guidelines identify regorafenib as the standard of care for HCC patients with advanced stage who have tolerated sorafenib but progressed. In the current study, treatment response of regorafenib with an ORR of 6.4% and a DCR of 31.9% was inferior to that of the RESORCE trial, but our median OS was equivalent (11 months *vs* 10.6 months).

In recent years, various systemic therapeutic options have been approved, so it is a critical issue for clinicians to decide on what is an appropriate second-line systemic treatment option after sorafenib failure? The current study compared the efficacy of nivolumab and regorafenib, the most frequently used ICI and TKI, for HCC patients with advanced stages where sorafenib treatment failed. In a mathematical Markov model reported by Cabibbo et al. that simulated treatment effect of sequential systemic therapies among patients with advanced HCC based on data of clinical trials, the simulated estimates of median OS were significantly higher for sofafenib followed by nivolumab compared to sorafenib followed by regorafenib (27 months *vs* 18 months) (19).

In the current study, we found that using nivolumab had a trend of better ORR and DCR than using regorafenib, but there was no statistical difference. Furthermore, median TTP and OS were not significantly different between the two groups in Kaplan-Meier survival analysis (TTP: 3 months *vs* 2.6 months, p = 0.786; OS: 14 months *vs* 11 months, p = 0.763 for the Nivolumab group *vs* Regorafenib group). Our finding is compatible to two previous Korean studies. Lee et al. reported that for 102 and 48 patients who were treated with nivolumab and regorafenib respectively, mOS was 5.9 and 6.9 months respectively (P = 0.77) (20). There was no obvious difference in DCR between nivolumab and regorafenib groups (50.0% *vs*. 47.1%; P =0.58). Another larger-sized study including 223 advanced HCC patients treated with regorafenib and 150 patients treated with nivolumab indicated that PFS (7.1 weeks for Nivolumab group *vs* 12 weeks for Regoranib group; P = 0.150), TTP (7.9 weeks *vs* 12.1 weeks; P = 0.680), and OS (32.6 weeks *vs* 30.9 weeks; P = 0.154) did not differ significantly between patients with nivolumab or regorafenib (21); however, the ORR was significantly higher in the Nivolumab vis-à-vis the Regorafenib group (13.3% *vs*. 4.0%; P = 0.002). It seems that the Nivolumab group might have superior treatment response, but clinical treatment outcomes such as TTP or OS might not be different.

The current study found that liver function reserve, achieved disease control and afforded post-treatment were independent factors associated with mortality for patients with advanced HCC receiving second-line treatment after sorafenib failure in multivariate analysis. However, using nivolumab or regorafenib was not related to overall survival, no matter for either univariate or multivariate analysis. Lee et al. reported that nivolumab was associated with prolonged OS (*vs*. regorafenib: HR, 0.54; 95% CI, 0.30–0.96; P =0.04) (20). However, in that study, OS did not differ in either group according to Kaplan Meier survival analysis (5.9 months in Nivolumab group *vs* 6.9 months in Regorafeib group, p=0.77) and univariate Cox regression analysis (Nivolumab (*vs*. regorafenib); HR: 1.081 (95%CI: 0.644–1.813) P=0.77). Choi et al. also reported that OS was consistent between these two groups by multivariable-adjusted, propensity score-matched and inverse probability treatment-weighted (IPTW) analyses (21). After cessation of regorafenib or nivolumab, the current study found 37.8% of patients (34.4% of Nivolumab group/39.7% of Regorafenib group) could afford following therapies. Patients with post-treatment had significantly superior median OS than those without (17.1 months *vs* 5.4 months, p<0.001), meaning that more than one-third of patients could maintain good liver function reserve and adequate performance status after experiencing nivolumab or regorafenib following sorafenib therapy. Regarding the impact of HCC etiology on treatment outcome, the current study found that hepatitis status was not associated with overall survival. Previous sub-analysis of CheckMate 040 study has indicated that the median OS of nivolumab in HCC patients with HBV or HCV was similar (22). In our Nivolumab group, patients with HBV or HCV also had insignificant median OS (6.3 months *vs* 10.3 months, p=0.885). Regarding the Regorafenib group, median OS from beginning sorafenib was almost statistically significant in HBV-related HCC or HCV-related HCC (18.9 months *vs* 14.2 months, p=0.051). This differs from the meta-analysis indicating that there is improved OS for patients negative for HBV and positive for HCV when treated with sorafenib (23).

Despite the fact that there was no OS difference between the Regorafenib group and the Nivolumab group, the two regimens really increased survival benefits for those patients who failed sorafenib treatment. Our previous study reported that the median OS of sorafenib use was only 8 months in the era of no effective sequential systemic therapies offered (24). The current study indicated overall OS from the beginning of sorafenib use was extended to 17.3 months in the Regorafenib group and 21.9 months in the Nivolumab group respectively. Consequently, application of regorafenib or nivolumab is approved as a potential second-line therapy followed sorafenib in clinical practice. Further well-designed prospectively randomized clinical trials are required to determine when and how to use regorafenib or nivolumab following sorafenib treatment for patients with unresectable HCC. Moreover, since resistance to targeted or ICI-based therapeutic drugs remains one of the main challenges for HCC treatment, other mechanisms blocking HCC cells such as poly (ADP-ribose) polymerase (PARP) inhibitor might be the future study frontier (25).

Concerning treatment safety, the current study found that the Regorafenib group had significantly higher proportions of TRAE than the Nivolumab group (68% *vs* 37.5%, p=0.006). The safety of regorafenib in the current study was demonstrated to be consistent with its safety profile in previous studies (6, 21, 23), with the leading four adverse events being HFSR (23.8%), diarrhea (11.9%), fatigue (10.2%) and elevated ALT (10.2%). Moreover, six patients (10.2%) had severe TRAE requiring permanent cessation of regorafenib, including one with HFSR, two with fatigue and three with hyperbilirubinemia. Compared with Regorafenib, the Nivolumab group had lower incidence of TRAE during treatment, with 37.5% including fatigue (12.1%), dermatitis (9.3%) and hyperbilirubinemia (6.2%). Only two patients (6.2%) had severe TRAE over grade 3, and both presented with hyperbilirubinemia. Although using regorafenib has more TRAE and poorer life quality than using nivolumab, the non-invasive oral-administered route and obviously cheaper price appear as advantages of regorafenib over nivolumab in real world consideration.

There are some limitations in the current study. Firstly, this was a retrospective study so that some biochemical and clinical data were not available at medical chart review. Approximately 20% of the patients that lacked image examinations following treatment might generate deviated assessments of treatment response. Secondly, in clinical real practice, baseline characteristics of Nivolumab and Regorafenib groups including liver function reserve and tumor pattern were not consistent, which might lead to confounding bias in the analysis. Thirdly, due to the small sample size of the enrolled patients, the analysis of TTP or OS might be affected by extreme values. Further large sample-sized studies are required to reduce these possible statistical biases.

In clinical practice, for patients with advanced HCC who failed sorafenib treatment, there was optimal survival benefit no matter whether using nivolumab or regorafenib as the second-line therapy. The Nivolumab group seemed to have lower TRAE incidence and a trend of better tumor response compared with the Regorafenib group; however, their TTP and OS did not differ significantly.

## Data Availability Statement

The original contributions presented in the study are included in the article/supplementary material. Further inquiries can be directed to the corresponding author.

## Ethics Statement

The studies involving human participants were reviewed and approved by Kaohsiung Chang Gung Memorial Hospital (IRB No: 202100227B0). Written informed consent for participation was not required for this study in accordance with the national legislation and the institutional requirements.

## Author Contributions

Y-HK and J-HW made substantial contributions to the study conception, design, analysis, and interpretation of the data. Y-HY, Y-YC, K-MK, C-HH, S-NL, T-HH, and C-HC contributed to the acquisition of the data. The first draft of the manuscript was written by Y-HK and J-HW. J-HW commented on subsequent versions of the manuscript. All authors approved the final manuscript submitted to the journal.

## Conflict of Interest

The authors declare that the research was conducted in the absence of any commercial or financial relationships that could be construed as a potential conflict of interest.
